# Intra-articular delivery of extracellular vesicles secreted by chondrogenic progenitor cells from MRL/MpJ superhealer mice enhances articular cartilage repair in a mouse injury model

**DOI:** 10.1186/s13287-020-01594-x

**Published:** 2020-03-02

**Authors:** Rikang Wang, Wei Jiang, Lang Zhang, Saisai Xie, Shuai Zhang, Shun Yuan, Yi Jin, Guangqian Zhou

**Affiliations:** 1 National Pharmaceutical Engineering Center for Solid Preparation in Chinese Herbal Medicine, Jiangxi University of Traditional Chinese Medicine, Nanchang, 330006 People’s Republic of China; 2 Department of Medical Cell Biology and Genetics, Shenzhen Key Laboratory for Anti-ageing and Regenerative Medicine and Guangdong Key Laboratory for Genome Stability and Disease Prevention, Health Science Center, Shenzhen University , Shenzhen, 518060 People’s Republic of China; 3 Jiangxi Provincial Children’s Hospital, Nanchang, 330006 People’s Republic of China

**Keywords:** Cartilage, Osteoarthritis, Proliferation, miRNA sequencing, Chondrogenic progenitor cells, MRL/MpJ mice

## Abstract

**Background:**

Chondrogenic progenitor cells (CPCs) have high self-renewal capacity and chondrogenic potential. Intra-articular delivery of purified mesenchymal stem cells (MSCs) from MRL/MpJ “superhealer” mice increased bone volume during repair and prevents post-traumatic arthritis. Recently, although extracellular vesicles released from MSCs have been used widely for treating OA, the application of extracellular vesicles secreted by CPCs from MRL/MpJ mice in OA therapy has never been reported. In this study, we evaluated the effects of extracellular vesicles secreted by CPCs from control CBA (CBA-EVs) and MRL/MpJ mice (MRL-EVs) on proliferation and migration of murine chondrocytes. We also determined here if weekly intra-articular injections of CBA-EVs and MRL-EVs would repair and regenerate surgically induced model in mice.

**Methods:**

CPC surface markers were detected by flow cytometry. CBA-EVs and MRL-EVs were isolated using an ultrafiltration method. Nanoparticle tracking analysis, transmission electron microscopy, and western blots were used to identify extracellular vesicles. CBA-EVs and MRL-EVs were injected intra-articularly in a mouse model of surgical destabilization of the medial meniscus (DMM)-induced OA, and histological and immunohistochemistry analyses were used to assess the efficacy of exosome injections. We used miRNA-seq analysis to analyze the expression profiles of exosomal miRNAs derived from CBA-EVs as well as MRL-EVs. Cell-counting and scratch assays were used to evaluate the effects of CBA-EVs and MRL-EVs on proliferation and migration of murine chondrocytes, respectively. Meanwhile, a specific RNA inhibitor assesses the roles of the candidate miRNAs in CPC-EV-induced regulation of function of chondrocytes.

**Results:**

Both CBA-EVs and MRL-EVs stimulated chondrocyte proliferation and migration, but MRL-EVs exerted a stronger effect than CBA-EVs. The similar result was also observed in in vivo study, which indicated that injecting either CBA-EVs or MRL-EVs attenuated OA, but MRL-EVs showed a superior therapeutic effect in comparison with CBA-EVs. The results of bioinformatics analyses revealed that the differentially expressed exosomal miRNAs participated in multiple biological processes. We identified 80 significantly upregulated and 100 downregulated miRNAs. Moreover, we found that the top 20 differentially expressed exosomal miRNAs connected OA repair to processes such as AMPK signaling, regulation of autophagy, and insulin signaling. Notably, miRNA 221-3p were highly enriched in MRL-Exos and treatment with miR 221-3p inhibitor markedly decreased chondrocyte proliferation and migration induced by CBA-EVs or MRL-EVs in vitro.

**Conclusions:**

This is the first study to demonstrate MRL-EVs had a greater therapeutic effect on the treatment of OA than CBA-EVs. This study will hopefully provide new insight into the pathogenesis, prevention, and treatment of OA.

## Introduction

The delivery of mesenchymal stem cells (MSCs) has been widely used as a regenerative therapy for various disease states. The progresses in stem cell transplantation therapy have become promising in the treatment of osteoarthritis (OA). In the past decade, adipose-derived MSCs (AMSCs) [1, 2] and bone marrow-derived MSCs (BMSCs) [3, 4] were successfully used in treating OA. The chondrogenic progenitor cells (CPCs) with MSC characteristics have been confirmed to have potential regenerative role in healthy and diseased articular cartilage tissue [5, 6]. As cartilage seed cells, CPCs are important to maintain cartilage homeostasis [6]. Moreover, CPCs might also play an important role in the manifestation of OA by reducing the proliferation and chondrogenesis in OA [7]. However, there are still many disadvantages in stem cell transplantation therapy that should be overcome, such as chromosomal variation and potential immunological rejection [8, 9]. Therefore, developing a superior strategy that can make full use of the advantages of stem cells without the potential risks of direct usage is highly important.

Recently, many studies have indicated that the activation of resident cells through a paracrine mechanism may be critical in progenitor cell-mediated tissue regeneration [10, 11]. Evidence has suggested that extracellular vesicles show similar biological function to the cells that they are derived from, and that direct use of these nanoparticles does not show any obvious adverse effects, such as tumorigenicity or immunogenicity [12]. There are various types of cells that can secrete extracellular vesicles. Recent studies have shown that bone-related cells, including osteoclasts, osteoclast precursors, osteoblasts, osteocytes, and MSCs, can also secrete extracellular vesicles [13]. As a cell-free MSC therapy, MSC exosome plays an important role in cartilage regeneration and osteoarthritis treatment [14–16].

The inbred MRL/MpJ mouse strain has shown remarkable regenerative capabilities after injury in different tissues including articular cartilage [17]. Intra-articular delivery of purified MSCs from “superhealer” MRL/MpJ mice increased bone volume during repairing and preventing post-traumatic arthritis [18]. Currently, whether extracellular vesicles secreted by CPCs from MRL/MpJ (MRL-EVs) or secreted by CPCs from control CBA mice (CBA-EVs) are better for the treatment of OA has not yet been reported. In this study, we compared the MRL-EVs and CBA-EVs for the treatment of OA.

## Materials and methods

### Cell isolation and culture

Fresh joints were obtained from CBA and MRL/MpJ mice (16 weeks old). According to a previously described method, articular cartilage was harvested and subjected to sequential trypsin/collagenase digestion to isolate CPCs and chondrocytes [19]. Briefly, the collected cartilage was digested in Dulbecco’s modified Eagle’s medium (DMEM)/F-12 medium containing 0.2% collagenase II (Life Technologies, CA, USA), 5% FBS (SeraBest, PAN-Systech GmbH, Germany), and 1% penicillin/streptomycin (Invitrogen) in a 37 °C shaker for 8 h. Then, the digested material was filtered through a 40-mm mesh sieve (BD Falcon, Heidelberg, Germany) to eliminate cell-matrix residues. The cells (10^3^/cm^2^) were transferred to 6 cm plastic dishes (10 μg/mL fibronectin coated overnight) and cultured in DMEM/F-12 with 15% FBS and 1% penicillin/streptomycin. Medium and non-adherent cells were removed after incubation for 20 min at 37 °C, and fresh culture medium was added to the dishes. The adherent cells were cultured until confluent for 7–10 days. Then, the colonies were collected and passaged two or three times prior to use in all experiments. At the same time, CPCs and chondrocytes were separated on the basis of CPCs’ differential adhesion to fibronectin, as described previously [19, 20]. The chondrocytes were supplemented with DMEM/F12 containing 1% penicillin/streptomycin and 10% FBS, and l-glutamine (4.5 mM) was used to expand the remaining separated cells. When cells reached 90% confluence, they were passaged using 0.25% trypsin.

### Characterization of CPCs

Surface antigens of CPCs were analyzed by flow cytometry as described previously [6]. Firstly, cells were harvested and incubated with 3% bovine serum albumin (BAS, Gibco) for 30 min in PBS to block non-specific antigen binding. Then, the CPCs were incubated with monoclonal antibodies against CD29, CD34, CD44, CD45, and CD90 (all antibodies from BD Biosciences, Sparks Glencoe, MD, USA). After that, the unbound antibody was removed by washing, and the surface antigens were analyzed using the Guava easyCyte™ flow cytometer (Millipore, Billerica, MA, USA).

### Extracellular vesicle isolation from CPCs

The CPCs were washed with PBS after they reach 80% confluence. Then, the culture medium was replaced, and the cells were incubated with complete DMEM/F-12 medium which contain extracellular vesicle-free FBS (Gibco, USA) for 48 h. Afterwards, the conditioned medium was collected and subjected to centrifuge at 300*g* for 10 min to eliminate cells and 2500*g* for 25 min to remove debris and apoptotic bodies. Then, 15 mL of supernatant was added to an Amicon Ultra-15 Centrifugal Filter Unit (100 kDa; Millipore) and centrifuged at 4000*g* to about 1 mL. The ultrafiltration liquid was centrifuged at 15,000*g* for 1 h in polyallomer tubes, and supernatant was filtered on 0.22 μm porous membrane and centrifuged at 110,000*g* for 2 h. Pellet was suspended in PBS and centrifuged at 110,000*g* for 2 h again. Exo pellets were suspended in 100 μL of PBS and freshly used for in vitro and in vivo functional experiments.

### Identification of extracellular vesicles

The size distribution of CBA-EVs and MRL-EVs was measured by nanoparticle tracking analysis with a Nanosight NS300 instrument (Malvern Instruments, Malvern, UK), and the extracellular vesicle morphologies were observed with a JEM-1400 transmission electron microscope (TEM) (Hitachi, Tokyo, Japan). The characteristics of exosomal surface marker proteins calnexin (1:1000; Abcam), CD9 (1:1000; Abcam), CD63(1:1000; Abcam), and TSG101 (1:1000; Abcam) were analyzed by western blots.

### Total RNA extraction and RNA sequencing

Total RNA was extracted from extracellular vesicles using the exoEasy Qiagen kit according to the manufacturer’s instructions. RNA samples were quantified by using a NanoDrop spectrophotometer (Thermo Scientific, MA, USA), and their quality was assessed on a bioanalyzer (Agilent Genomics, CA, USA).

The miRNA sequencing library was prepared by extracting total RNA from each sample. Firstly, the RNA molecules in a size range of 18–30 nt were enriched by polyacrylamide gel electrophoresis (PAGE). Then, the 3′ adapters were added and the 36–44 nt RNAs were enriched. After that, the 5′ adapters were ligated to the RNAs as well. The ligation products were reverse transcripted by PCR amplification, and the 140–160 bp size PCR products were enriched to generate a cDNA library and sequenced using Illumina HiSeqTM 2500 by Sagene Biotech Co. Ltd (Guangzhou, China).

### miRNA-seq data analysis

After sequencing, Solexa CHASTITY QC was performed to collect filtered raw reads as clean reads. The adaptor sequences were trimmed, and the adaptor-trimmed reads (≥ 15 nt) were left. GenBank database (release 209.0) and Rfam database (11.0) were used to identify and remove rRNA, scRNA, snoRNA, snRNA, and tRNA. miRBase database (release 21) was used to identify known miRNAs (exist miRNAs). Mireap_v0.2 was applied to identify the novel miRNA candidates. The most abundant isomiR, the mature miRNA annotated in miRBase, and all isoforms of the miRNA (5p or 3p) were used to calculate miRNA expression. The significant differentially expressed miRNAs were identified by fold change and *p* value, which were calculated from comparing the differentially expressed miRNA profiles between the two groups. Differentially expressed miRNAs between two samples were filtered by fold change. Hierarchical clustering was performed. Two database, TargetScan 7.1, RNAhybrid (v2.1.2)+svm_light (v6.01), and Miranda(v3.3a) were performed to predict miRNA target. The overlapping results of three databases were miRNA targets. Cytoscape software was carried out to obtain the network of miRNAs and mRNAs, showing the relationship between miRNAs and targets. The DAVID (version 6.7, https://david-d.ncifcrf.gov/) web webserver was used to perform enrichment analysis of top 10 differentially expressed miRNAs in Gene Ontology (GO) and Kyoto Encyclopedia of Genes and Genomes (KEGG) pathway. Gene lists were uploaded to DAVID with “Official Gene Symbol” as identifier and submitted to analysis using the whole human genome as background. The *p* value denotes the significance of GO term enrichment in the DE genes. Then, Benjamini and Hochberg method was applied to adjust the *p* value in order to avoid false positives due to chance, and *p* value less than 0.05 was considered to be significantly enriched.

### Extracellular vesicle uptake by chondrocytes

To determine extracellular vesicle uptake by chondrocytes, extracellular vesicles were labeled with a PKH26 staining kit (Sigma-Aldrich, St. Louis, MO, USA) according to a previously reported procedure [21]. The EV-labeled suspension was centrifuged at 110,000×*g* for 2 h, and the supernatant was discarded. Then, EVs were resuspended in PBS (10^8^ particles/mL) and incubated with chondrocyte at 37 °C for 3 h. When the incubation was completed, the chondrocyte was washed with PBS and fixed with 4% paraformaldehyde for 15 min. After this, nuclei were stained with DAPI (0.5 μg/mL; Invitrogen, Carlsbad, USA). A fluorescence microscope (Leica DMI6000B, Solms, Germany) was used to determine the fluorescence in chondrocyte.

### Proliferation assay

Cell proliferation was evaluated using a cell counting kit-8 assay (CCK-8; Dojindo, Kyushu Island, Japan) according to the manufacturer’s instructions. Briefly, cells were seeded in a 96-well plate at a density of 5000 cells per well and grown overnight at 37 °C in a humidified incubator with 5% CO_2_. Then, cells were treated with PBS or extracellular vesicles (10^8^ particles/mL) from different groups. After incubation for 24 h, 48 h, and 72 h, 10 μL of CCK-8 solution and 100 μL of fresh culture medium were added to each well and incubated at 37 °C for 1 h. The optical density (OD) was determined at 450 nm with an automated microplate reader (Bio-Rad 680, Bio-Rad, Hercules, CA, USA).

### Chondrocyte migration assay

The effect of extracellular vesicles on migration of chondrocytes was analyzed by scratch wound assay as previously described [16]. Briefly, chondrocytes were seeded into 12-well plates at density of 15,000 per well and maintained at 37 °C for 8 h. Next, the confluent monolayer of cells was scratched with a P200 pipet tip and washed with PBS to remove floating cells. Then, the medium was replaced with fresh DMEM/F-12 medium containing control medium, 10^8^ particles/mL CBA-EVs or 10^8^ particles /mL MRL-EVs. The rate of migration area was photographed by collecting digital images at 0, 24, and 48 h after the scratch using an inverted microscope (Leica, Wetzlar, Germany). Migration area (%) = (*A*_0_ − *A*_*n*_)/*A*_0_ × 100, where *A*_0_ represents the area of initial wound and *A*_*n*_ represents the remaining area of wound at the metering point.

### RNA interference

MiR-221-3p inhibitors and their negative control inhibitors were purchased from RiboBio (Guangzhou, China). Cell transfection was performed following the handbook from RiboBio. Briefly, the cells were cultured in 6-well culture plate and transfected with miR-221-3p inhibitor or the negative control inhibitor using Lipofectamine 3000 (Invitrogen), and cultured in complete medium containing CBA-EVs or MRL-EVs (10^8^ particles/mL) or an equal volume of PBS. After 48 h of incubation, the downstream experiments were performed.

### Surgical-induced OA model

Mice were group housed (six mice per cage) in colony cages under a standard 12-h light/dark cycle with free access to water, standard mouse chow, and running wheels. All animal experiments were performed in agreement with the institutional guidelines for animal care and use and approved by the Animal Research Committee of Shenzhen University (approval number: SYXK2011-0128). Eight-week-old female C57B/L10 mice were divided randomly into four groups: CBA-EV treatment (*n* = 10), MRL-EV treatment (*n* = 10), OA (*n* = 10), and normal (without surgery; *n* = 10). On day 0, all mice in the CBA-EV, MRL-EV, and OA treatment groups were treated with surgical destabilization of the medial meniscus (DMM) to induce OA [22]. On the first day of every week from the fourth to the seventh week after surgery, mice in the CBA-EV and MRL-EV treatment groups were injected intra-articularly with 8 μL CBA-EVs (10^10^ particles/mL) or 8 μL MRL-EVs (10^10^ particles/mL) in PBS, respectively. Mice in the OA and normal groups were injected intra-articularly with 8 μL PBS at each time point. Mice were euthanized at 8 weeks post-surgery for preparation of paraffin sections and subsequent histological analysis.

### Histology and immunohistochemistry analysis

After harvesting the knee samples, the tibiofemoral joints were fixed in 10% paraformaldehyde for 24 h. Then, the fixed femoral condyles were decalcified in 10% EDTA for 7 days at 37 °C. After graded ethanol dehydration, vitrification with dimethylbenzene, and embedding in paraffin wax, the processed femoral condyles were sectioned at 5 μm thickness and stained with toluidine blue staining and safranin O/fast green. Osteoarthritis Research Society International (OARSI) score (a well-recognized histological scoring system) was used to evaluate the OA progress of every sample in each group [23].

The function of articular chondrocytes was evaluated by immunohistochemistry. Sections were incubated with primary antibodies (all from Abcam, 1:100) against anti-collagen I, anti-collagen II, and anti-aggrecan antibodies overnight at 4 °C. Biotinylated secondary antibody and streptavidin peroxidase solution were then used to visualize the sections.

### Quantitative real-time PCR analysis

Exosomal miRNAs were extracted using TRNzol Universal Reagent (Tiangen Biotech, Beijing, China), and cDNA for miRNAs was synthesized by the Bulge-LoopTM miRNA qRT-PCR Starter Kit (RiboBio, Guangzhou, China) according to the manufacturer’s protocol. The qRT-PCR reaction was also performed using Bulge-LoopTM miRNA qRT-PCR Starter Kit (RiboBio, Guangzhou, China) with the miRNA-specific forward primer and the universal reverse primer (RiboBio, Guangzhou, China). U6 small nuclear RNA was used to normalize the results.

### Statistical analysis

All results are represented as means ± SEM for 3–5 experiments. Differences between groups were analyzed using ANOVA, followed by the Tukey-Kramer or Dunnett’s multicomparison test with SPSS Software (SPSS Inc., Chicago, IL, USA). *p* < 0.05 was considered statistically significant.

## Results

### Identification of CPCs and CPC-derived extracellular vesicles

**Fig. 1:**
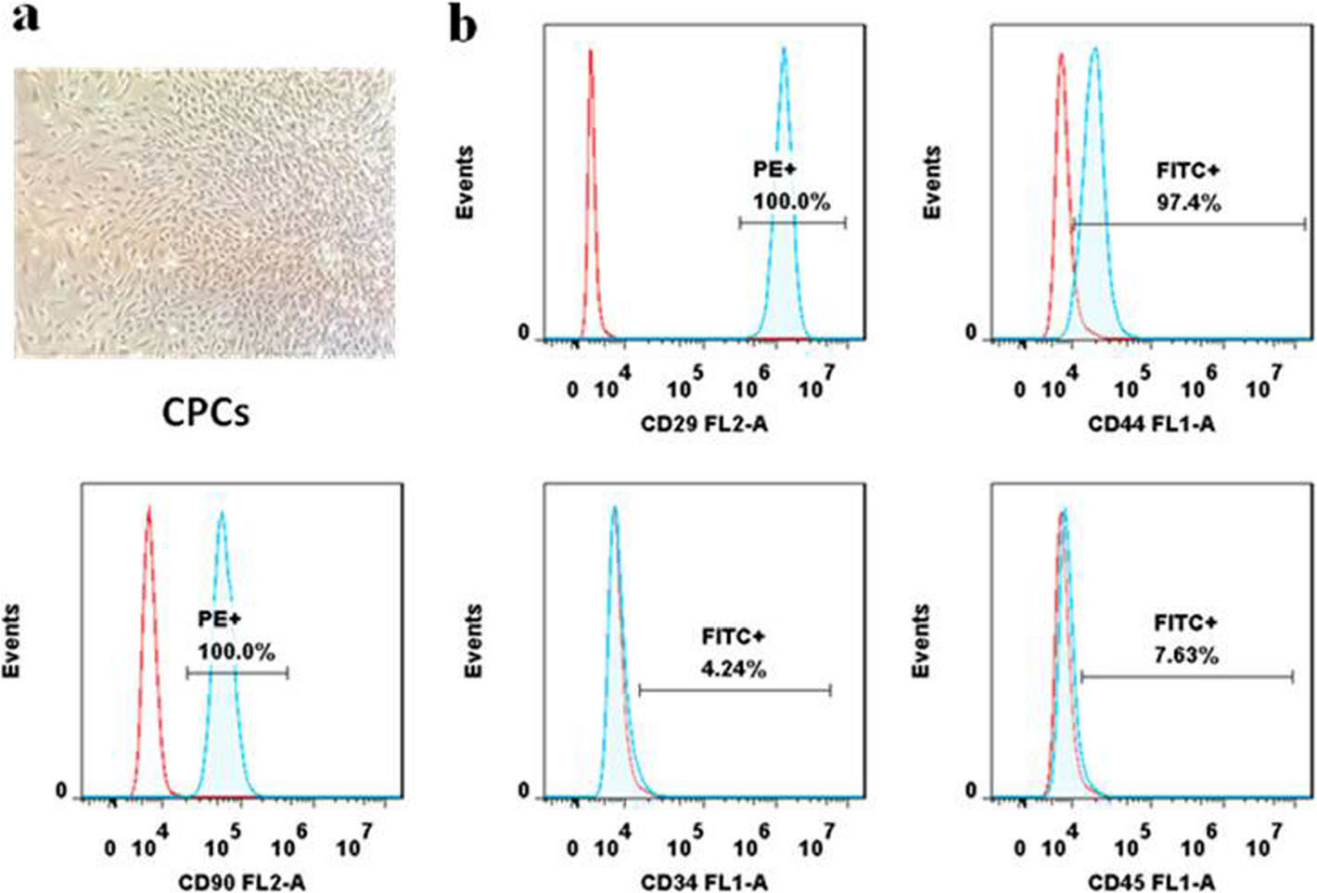
Identification of CPCs. **a** CPCs showed a representative spindle-like morphology. **b** Flow cytometric analyses of cell surface markers on CPCs. The isotype controls are illustrated as blank curves, and the test samples are illustrated as solid blue curves. CPCs were positive for CD29, CD44, and CD90 and were negative for CD34 and CD45

It has been reported that CPCs are characterized with clonogenicity, multipotency, and self-renewal capacity [24]. We isolated CPCs from the knee joint cartilage of CBA and MRL/MpJ mice using a monoclonal method. Single-cell suspensions from cartilage tissues were generated and cultured for 5–7 days. As shown in Fig. 1a, colonies were formed and exhibited a spindle-like morphology. Flow cytometry analysis demonstrated that the majority of CPCs expressed MSC surface markers including CD29, CD44, and CD90, but negative for CD34 and CD45 (Fig. 1b). All these features were consistent with previous studies [6, 25]. Moreover, our previous studies had confirmed that CPCs had the multilineage differentiation potential [26].

**Fig. 2:**
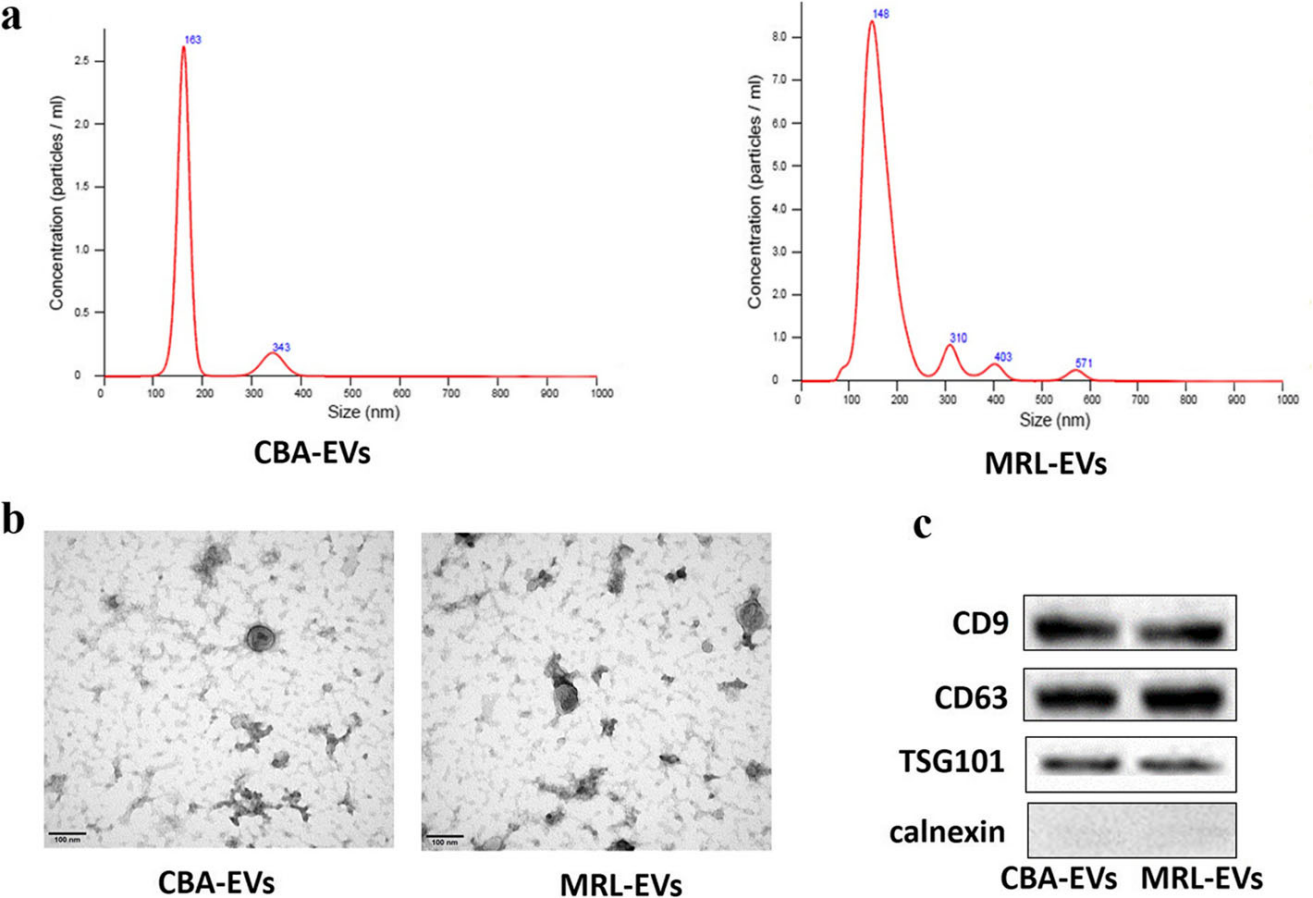
**a** Particle size distribution of extracellular vesicles determined by NTA. **b** Morphology of extracellular vesicles under TEM. Scale bar, 100 nm. **c** Exosome-specific CD9, CD63, TSG101, and calnexin proteins measured using western blotting. CBA-EVs, extracellular vesicles derived from CPCs of CBA mice; MRL-EVs, extracellular vesicles derived from CPCs of MRL/MpJ mice

Nanoparticle tracking analysis (NTA) indicated that the diameters of the majority of CBA-EVs and MRL-EVs ranged from 100 to 200 nm (Fig. 2a). Transmission electron microscopy (TEM) revealed that extracellular vesicles showed a cup- or sphere-shaped morphology with a diameter of 50–150 nm (Fig. 2b). Western blot analysis showed the CBA-EVs and MRL-EVs expressed exosomal surface markers including CD9, CD63, and TSG101 proteins (Fig. 2c).

### Effect of CBA-EVs and MRL-EVs on an OA mice model

**Fig. 3:**
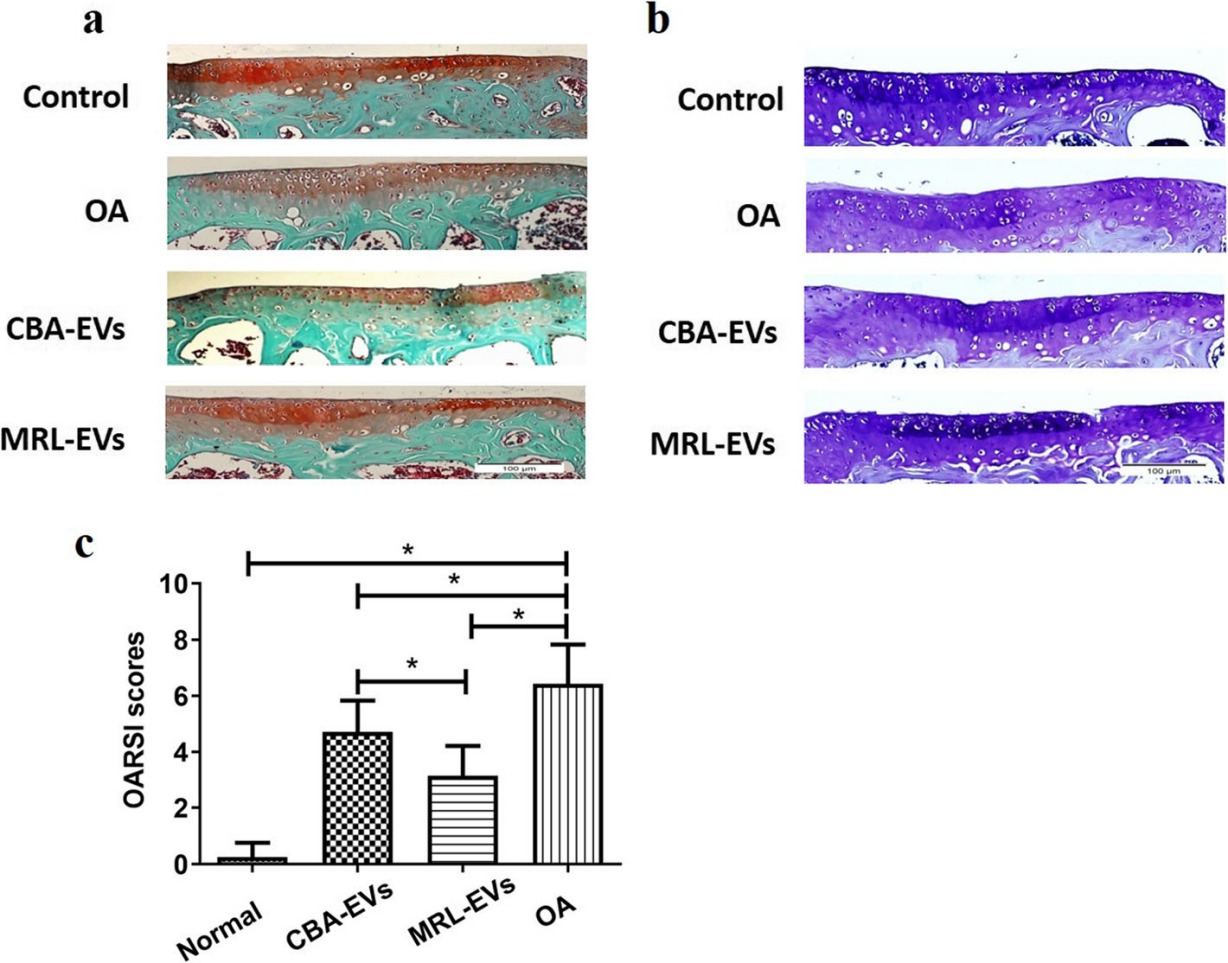
Histological analysis. **a** Representative safranin O/fast green staining micrographs display histopathological changes. **b** Representative toluidine blue staining micrographs display histopathological changes. **c** Statistical analysis of OARSI score in each group. OARSI scores in the normal, CBA-EV, and MRL-EV groups were significantly lower than in the OA group. The score of the MRL-EV group was significantly lower than that of the CBA-EV group, *p< 0.05. OA, osteoarthritis; OARSI, Osteoarthritis Research Society International. *n* = 10 per group

Safranin-O/fast green-stained sections were performed to assess matrix proteoglycan and overall joint morphology [27]. In the present studies, histological assessment with safranin O staining indicated that either CBA-EV or MRL-EV group showed much denser metachromatic staining in comparison with OA. Compared with the MRL-EV group, the CBA-EV group showed a loss of proteoglycan in cartilage (Fig. 3a). The same results were also shown in toluidine blue staining. The intensity of cartilage toluidine blue staining tended to increase in the CBA-EVs and MRL-EVs, but decrease in the OA model, which was primarily due to the loss of stain in the area of central erosion. (Fig. 3b). The OARSI scores were significantly lower in the normal, CBA-EV, and MRL-EV groups than in the OA group (Fig. 3c). The score in the CBA-EV group was significantly higher than that in the MRL-EV group.

**Fig. 4:**
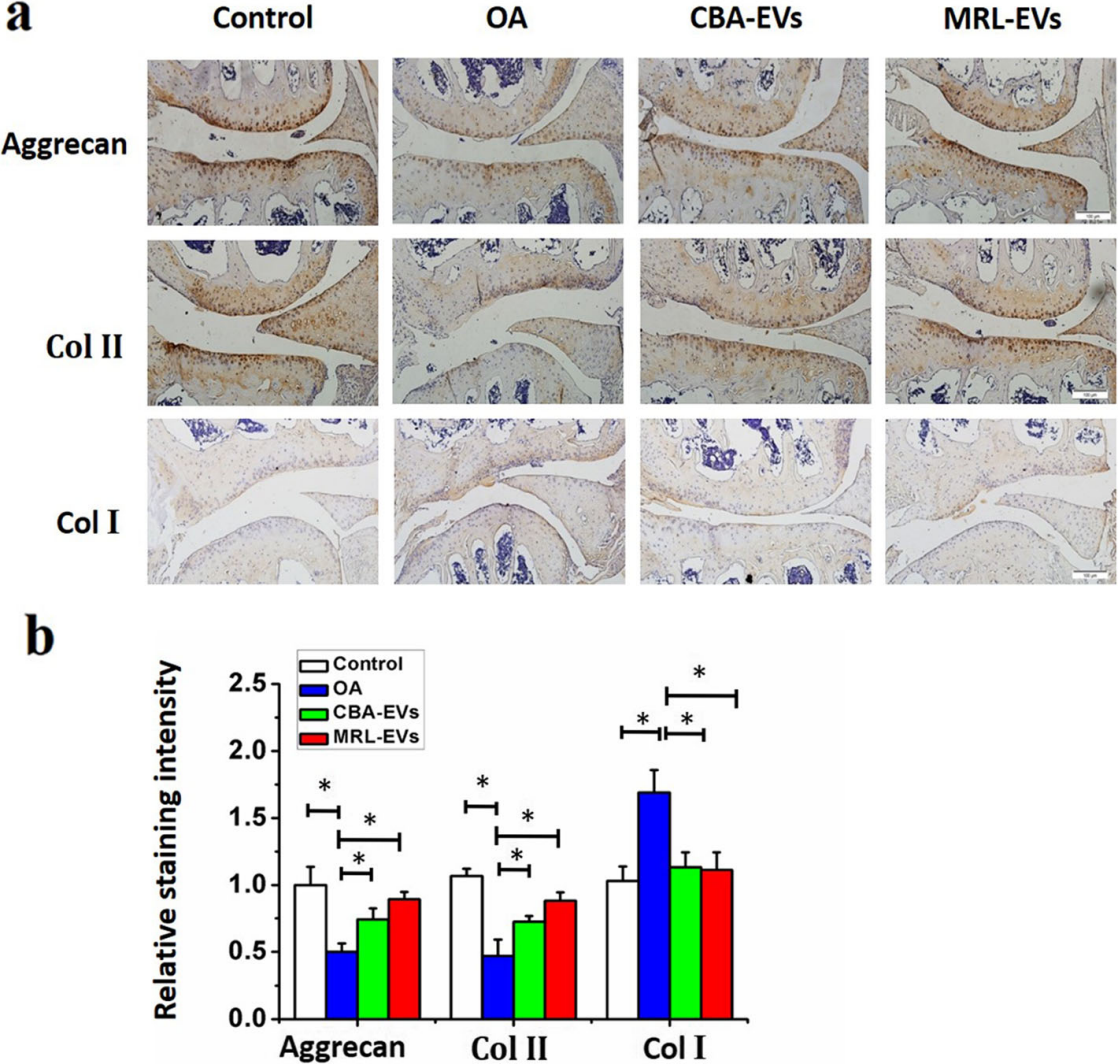
Immunohistochemical staining for type II collagen, type I collagen, and aggrecan (*n* = 10 for each group). **a** Photomicrographs of joint sections stained using anti-type II collagen, anti-aggrecan, or anti-type I collagen as primary antibodies (scale bar, 50 μm). **b** Quantification of aggrecan, Col II, and Col I in cartilage samples, *p < 0.05. Col II, type II collagen; Col I, type I collagen. *n* = 6 per group

IHC analysis of articular cartilage indicated that severe joint wear and cartilage matrix loss were observed in the OA group. Moreover, the expression of type II collagen (Col II) and aggrecan in cartilage was also decreased in the OA group. Collagen type I (Col I) staining was observed on the cartilage surface of the OA group, but it showed weak expression in the normal, CBA-EV, and MRL-EV groups. Compared to the OA group, intra-articular injection of CBA-EVs or MRL-EVs decreased the expression of Col I but increased aggrecan and Col II in the cartilage matrix. Moreover, the joint wear and cartilage matrix loss were much less severe in the CBA-EV or MRL-EV group. In the MRL-EV group, although joint wear was still present, the severity was relatively mild. The expression of Col II was slightly thinner than in the normal group, but it was significantly better than in the OA group or the CBA-EV group (Fig. 4).

### miRNA profiling of extracellular vesicles secreted by CPCs from CBA and MRL/MpJ mice

**Fig. 5:**
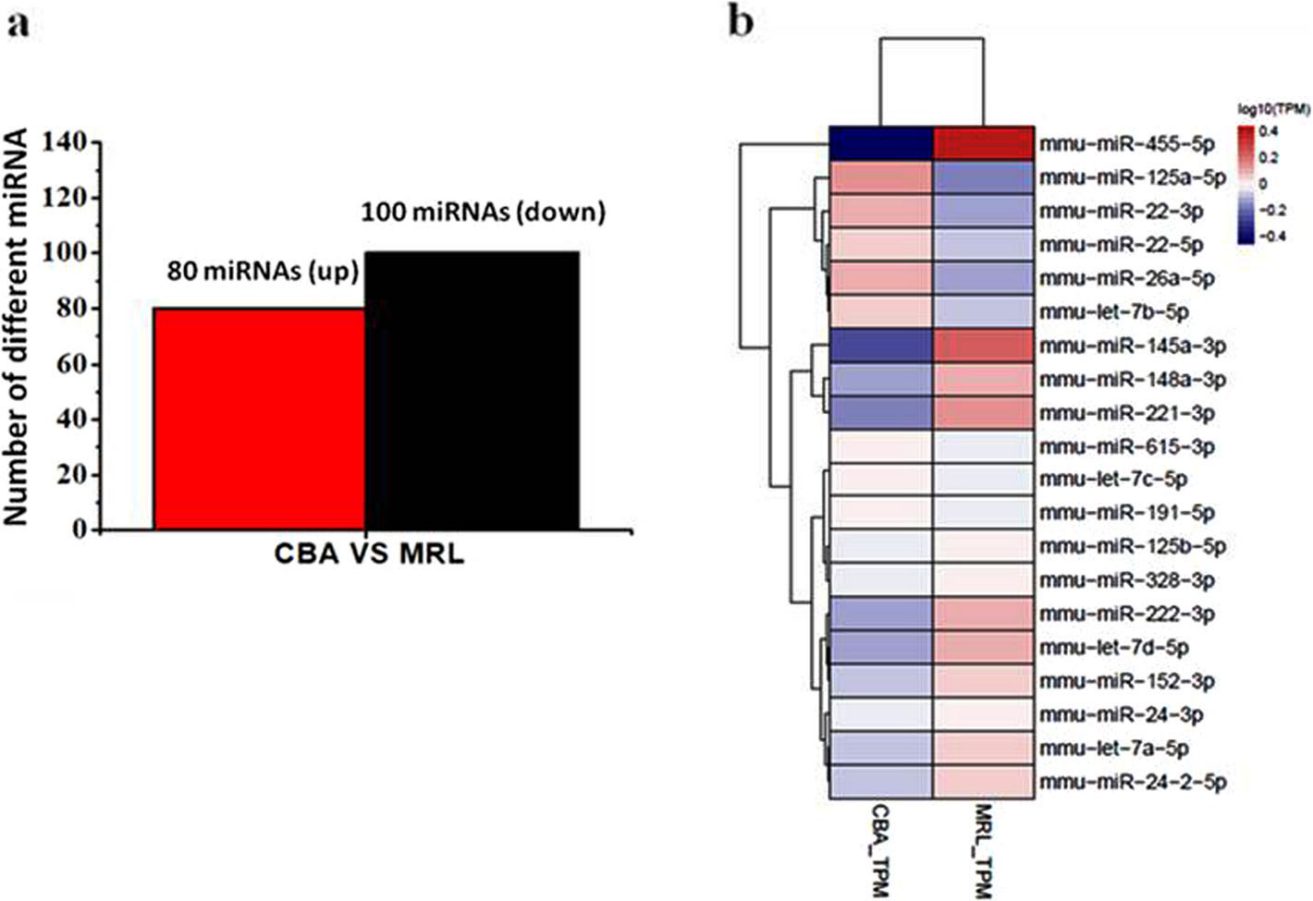
The miRNA differential expression profiles in EVs. **a** The histogram showed the differentially expressed miRNAs in CBA-EVs and MRL-EVs. Compared with CBA-EVs, the red histogram represent upregulation for the MRL-EVs, and the black histogram represent downregulation of expression for the MRL-EVs. **b** The heatmap of top 20 differentially expressed miRNAs in CBA-EVs and MRL-EVs based on miRNA sequence analysis

To understand the underlying mechanisms that how extracellular vesicles promoted chondrogenesis, we identify differentially expressed miRNAs of CBA-EVs and MRL-EVs by RNA sequencing. We detected a total of 191 miRNAs expressed in CBA-EVs or MRL-EVs. Compared with CBA-EVs, 80 miRNAs were upregulated and 100 miRNAs were downregulated in MRL-EVs (Fig. 5a). The top 20 differentially expressed miRNAs (let-7c-5p, miR-26a-5p, miR-148a-3p, let-7b-5p, miR-22-3p, miR-24-3p, let-7a-5p, miR-125b-5p, miR-615-3p, let-7d-5p, miR-125a-5p, miR-191-5p, miR-221-3p, miR-222-3p, miR-152-3p, miR-328-3p, miR-24-2-5p, miR-145a-3p, miR-22-5p, and miR-455-5p) account for more than 30% of total miRNAs present in CPC extracellular vesicles (Fig. 5b), and the top 10 upregulated miRNAs and downregulated miRNAs with fold change ≥ 1.5 (or ≤ 0.67) are miR-26a-5p (down), miR-148a-3p (up), let-7b-5p (down), miR-22-3p (down), let-7d-5p (up), miR-125a-5p (down), miR-221-3p (up), miR-222-3p (up), miR-145a-3p (up), and miR-455-5p (up).

### Pathway analysis and functional analysis of exosomal miRNAs

**Fig. 6:**
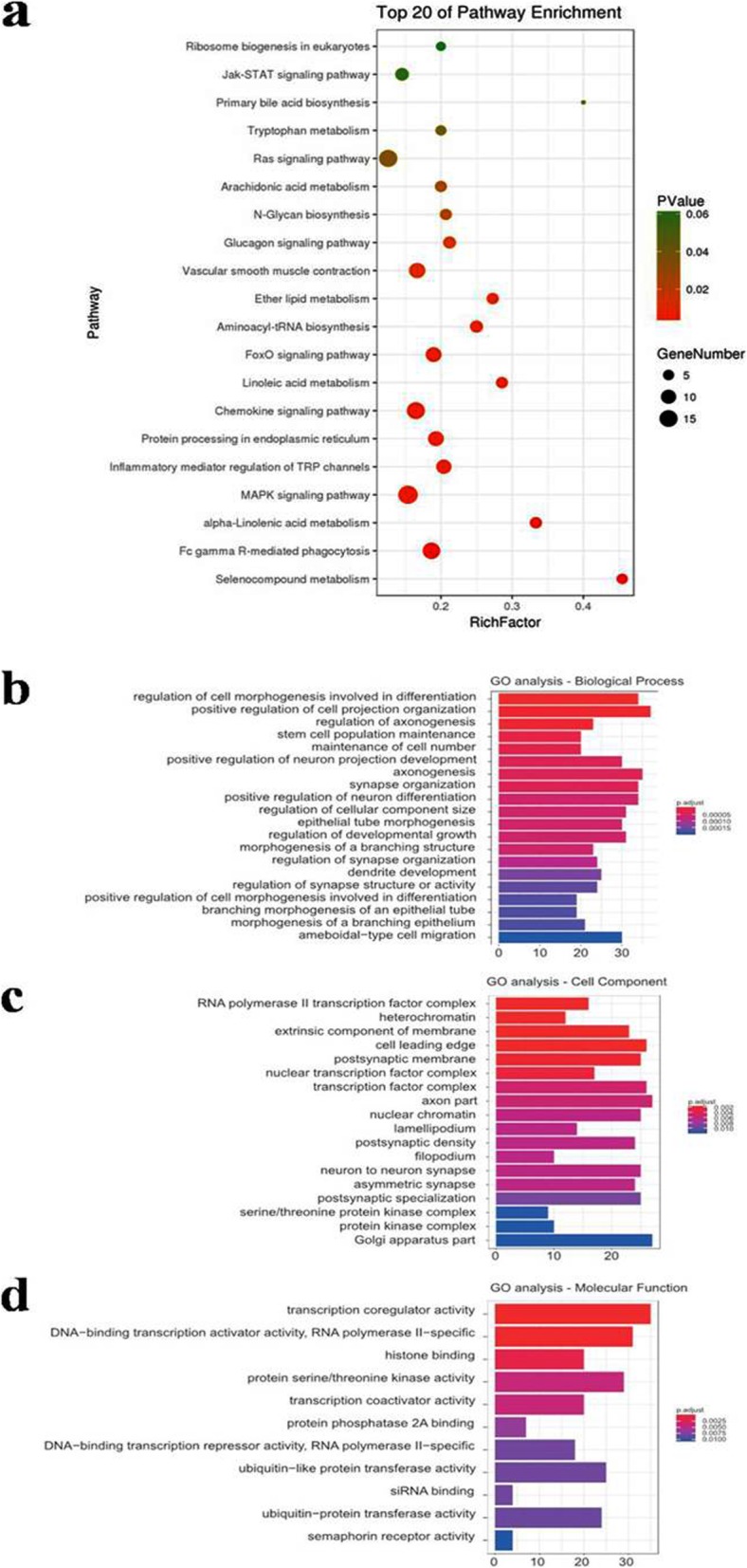
Pathway analysis and GO analyses of the targeted genes of the top 20 upregulated and downregulated miRNAs. **a** Enrichment map of KEGG pathway analysis. **b** Enrichment map of GO analyses—biological process analysis. **c** Enrichment map of GO analyses—cellular component analysis. **d** Enrichment map of GO analyses—molecular function. KEGG, Kyoto Encyclopedia of Genes and Genomes pathway analysis; GO, Gene Ontology

The top 20 differentially expressed miRNAs were selected for GO and KEGG enrichment analysis. The results revealed that the target genes of the differentially expressed miRNAs are principally associated with the processes, such as the Ras signaling pathway (*p* value = 0.0349), FoxO signaling (*p* value = 0.0043), chemokine signaling pathway (*p* value = 0.0038), inflammatory mediator regulation of TRP channels (*p* value = 0.0036), and MAPK signaling pathway (*p* value = 0.0034) (Fig. 6a). It suggested that these biologic pathways were involved in chondrogenesis and inflammatory regulation. To explore the potential biological functions of the top 20 differentially expressed miRNAs, GO enrichment analyses, including biological process, cellular component, and molecular function, were performed by clusterProfiler. According to the statistically significant GO analysis results (*p* values < 0.05), we found that the functions, such as regulation of cell morphogenesis involved in differentiation (*p* value = 6.27E−11), positive regulation of cell projection organization (*p* value = 6.16E−09), stem cell population maintenance (*p* value = 2.53E−08), maintenance of cell number (*p* value = 3.80E−08), regulation of developmental growth (*p* value = 1.89E−07), and positive regulation of cell morphogenesis involved in differentiation (*p* value = 6.52E−07), are mainly affected by differentially expressed miRNAs (Fig. 6b–d). Interestingly, these functions were closely related to chondrocyte proliferation and migration.

### miRNA-mRNA network analysis

**Fig. 7:**
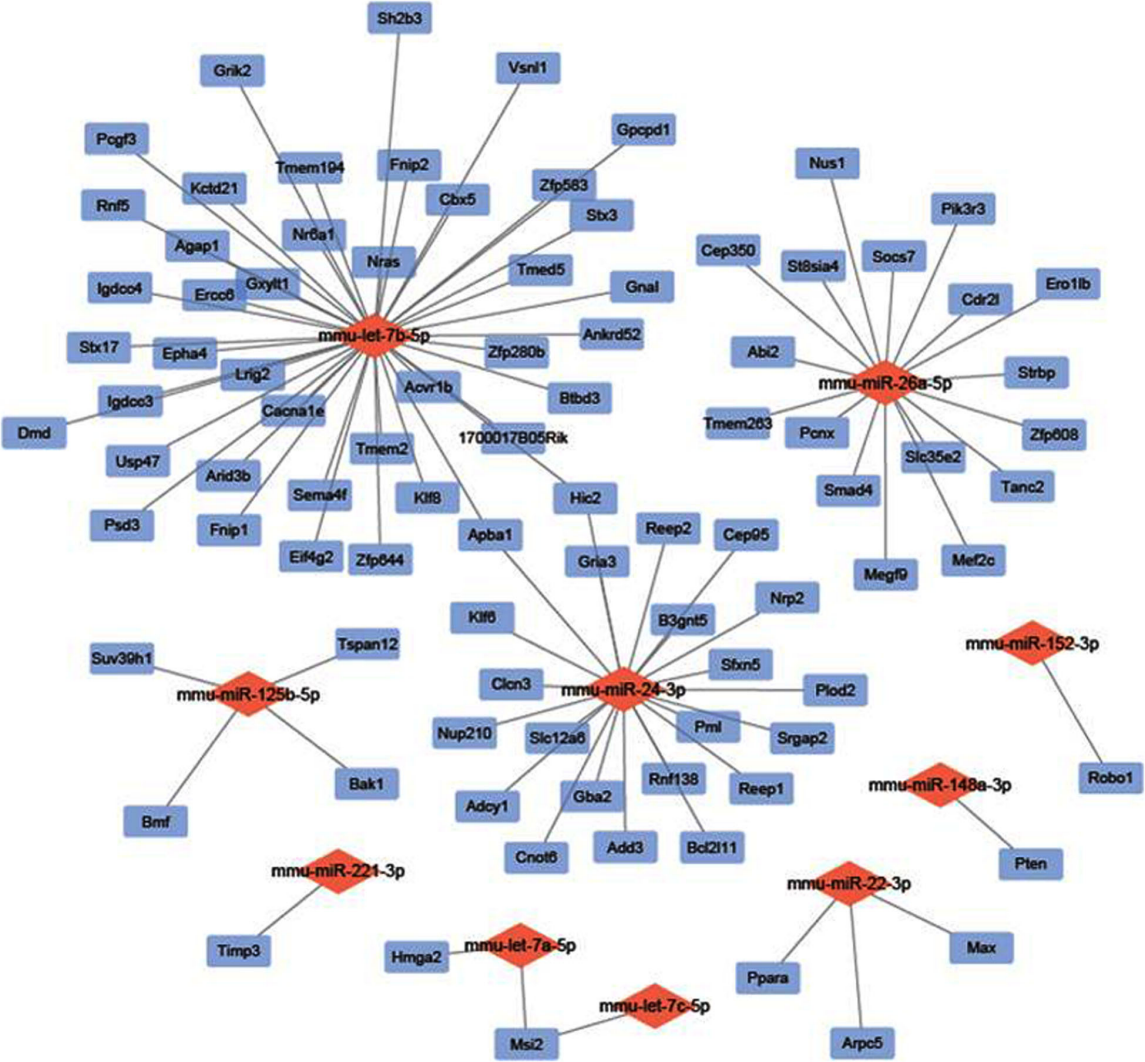
The relationship between differentially expressed miRNAs and predicted downstream mRNA is clearly shown in the miRNA-mRNA network. Blue, potential targeted gene; red, miRNA

We chose the top 20 differentially expressed miRNAs to predict downstream target genes using TargetScan, miRTarBase, miRDB, and constructed miRNA-mRNA networks using Cytoscape. We discovered that 8 miRNAs may recognize multiple target mRNAs simultaneously, and 1 gene may also be regulated by multiple miRNAs. More importantly, a great quantity of miRNAs was predicted to be involved in a variety of pathways that promote cartilage regeneration. The targeted genes and networks are shown in Fig. 7.

### Validation of miRNA expression by qPCR

**Fig. 8:**
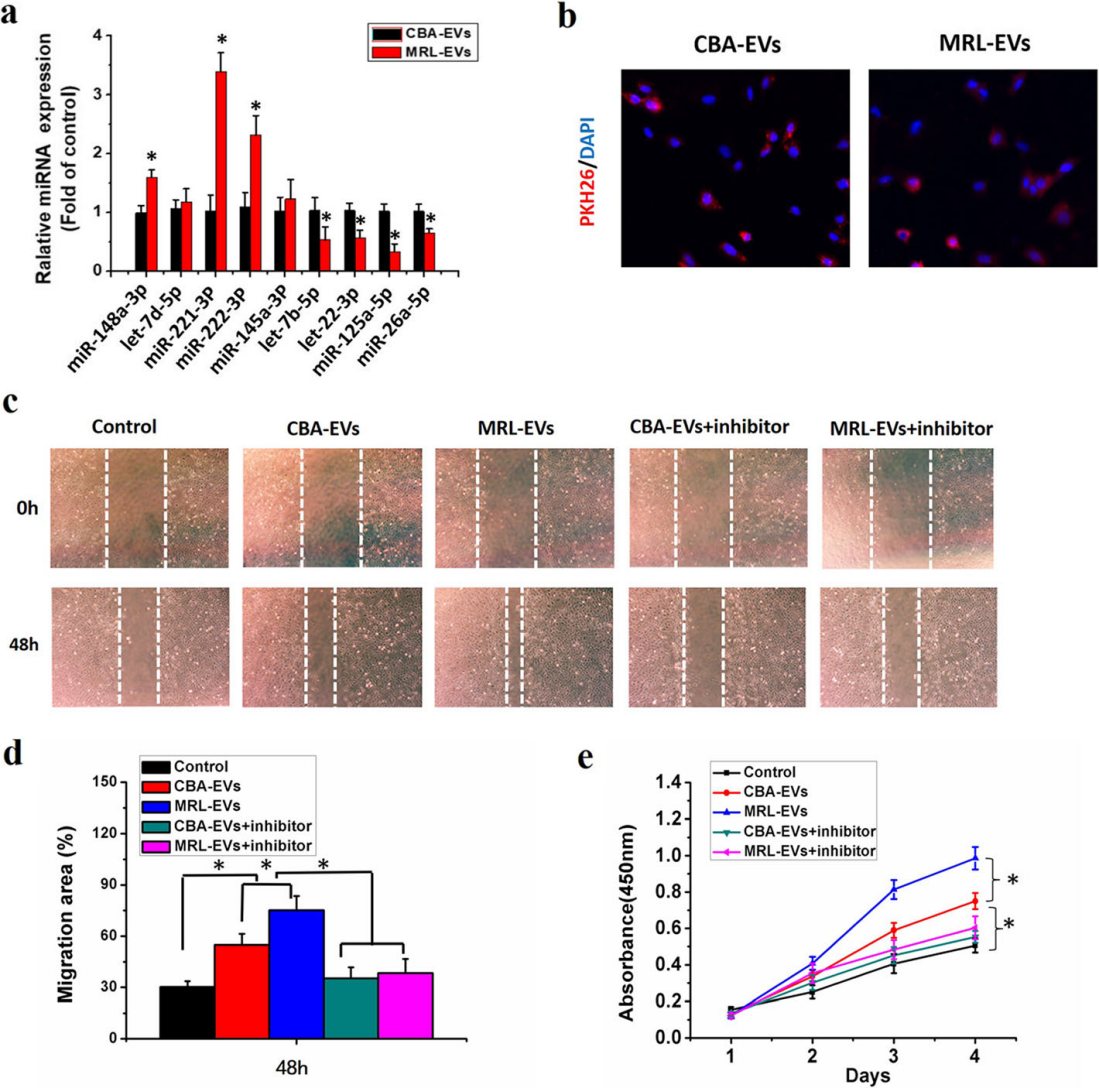
MRL-EVs promote the chondrogenesis via transferring miR-221-3p. **a** Quantitative real-time PCR validation of miRNAs in extracellular vesicles secreted by CPCs derived from CBA and MRL mice. *p < 0.05 represents significant differences between CBA-EVs and MRL-EVs. *n* = 3 per group. **b** Representative immunofluorescence photomicrograph of PKH26 (red)-labeled extracellular vesicles absorbed by chondrocytes, the nuclei of which were stained by DAPI (blue). **c** Light microscopy images of scratch wound assays. The migration of chondrocytes in different treatment groups was tested by the scratch wound assay. **d** Quantitative analysis of migration rates at 48 h. Both CBA-EVs and MRL-EVs promoted chondrocyte migration and that MRL-EVs were more effective than CBA-EVs, but this effect was reduced by miR-221-3p inhibitor. *p < 0.05. *n* = 3 per group. **e** Effects of CBA-EVs and MRL-EVs on proliferation of chondrocytes. CCK-8 assay showed that CBA-EVs and MRL-EVs promoted chondrocyte proliferation and that MRL-EVs were more effective than CBA-EVs, but the miR-221-3p inhibitor markedly decreased their upregulation induced by CBA/MRL-EVs. *p < 0.05. *n* = 3 per group

We chose 9 miRNAs (miR-148a-3p, let-7d-5p, miR-221-3p, miR-222-3p, miR-145a-3p, let-7b-5p, miR-22-3p, miR-125a-5p, miR-26a-5p) with the most obvious differential expression to validate the results of miRNA-seq analysis using qPCR. Consistent with the miRNA-seq data, compared with extracellular vesicles derived from CBA-CPCs, the expression of 3 miRNAs [miR-148a-3p (up), miR-221-3p (up), miR-222-3p (up)] in MRL-EVs was significantly increased (*p* < 0.05), while the expression of let-7b-5p (down), miR-22-3p (down), miR-125a-5p (down), and miR-26a-5p (down) was significantly decreased (*p* < 0.05, Fig. 8a). But there were no significant differences for the expression of let-7d-5p and miR-145a-3p between CBA-EVs and MRL-EVs from the qPCR results (*p* > 0.05).

### Chondrocyte migration and proliferation assays

We firstly investigated whether CBA-EVs and MRL-EVs could be transferred into chondrocytes. It can be seen from Fig. 8b that the red fluorescent dye (PKH26)-labeled EVs were incorporated into the chondrocytes after 3 h incubation.

To determine the impact of CPC-derived extracellular vesicles (CPC-EVs) on chondrocyte function, chondrocytes were treated with CBA-EVs or MRL-EVs (10^8^ extracellular vesicles/mL) for the indicated times. Scratch wound assays revealed that both MRL-EVs and CBA-EVs led to a remarkable increase in chondrocyte migration in comparison with the control groups. Moreover, MRL-EVs showed more effective ability to increase the motility than CBA-EVs at 48 h, but the miR-221-3p inhibitor markedly decreased their upregulation induced by CBA-EVs or MRL-EVs (Fig. 8c, d). CCK-8 assay suggested that chondrocytes exhibited a much higher proliferative ability when they were cultured with CBA-EVs or MRL-EVs for 3 days (Fig. 8e). Interestingly, MRL-EVs showed a much stronger effect on chondrocyte proliferation than CBA-EVs, whereas this effect was attenuated by miR-221-3p inhibition.

## Discussion

Data support that CPCs have high self-renewal capacity and chondrogenic potential, and have a central role in cartilage tissue in health and disease. Thus, intrinsic regeneration capacity mediated by CPCs may be critical to cartilage degeneration and regeneration [28]. Previously, our and other lab have successfully identified, isolated, and characterized cells with stem or progenitor properties in the articular cartilage of mice models [26, 29, 30]. CPCs exhibited stem cell characteristics, such as multipotency, clonogenicity, and migratory activity. There is also evidence that MRL/MpJ mice have reduced inflammation, which may play a role in protecting these mice from PTOA [31]. Previous studies have shown that the repair of cartilage defects was enhanced in MRL/MpJ mice as compared to that in B6 mice [17]. However, in another study, it was found that MSCs derived from MRL/MpJ mice did not enhance articular cartilage repair compared to MSCs derived from C57BL/6 J control mice, although both MSCs have beneficial effects when injected into an injured joint [32]. Intra-articular delivery of stem cells to the knee has led to some engraftment in joint structures [33, 34], which indicated that while macrophages and other immune cells may be important to MRL/MpJ tissue regeneration, they are not sufficient to induce tissue repair in the absence of MSCs. Extracellular vesicles had no obvious immunogenicity and had the biological function similar to the cells from which they are derived [15]. Recent studies have shown that paracrine mechanisms including extracellular vesicles are responsible for stem cell- or progenitor cell-mediated tissue regeneration [35, 36]. However, there no reports with regard to the application of extracellular vesicles derived from CPCs in OA therapy, and whether extracellular vesicles secreted by CPCs from MRL/MPJ mice are more beneficial for the treatment of OA, which has not yet been reported.

In the present study, we explored the differences between the effect of CBA-EVs and MRL-EVs on the chondrogenesis and on the treatment of OA. Our results indicated that both CBA-EVs and MRL-EVs stimulated chondrocyte proliferation and migration, and MRL-EVs had a greater effect than CBA-EVs. Further in vivo study indicated that the injection of either CBA-EVs or MRL-EVs attenuated OA in a mouse DMM-induced OA model, but MRL-EVs had a superior therapeutic effect in comparison with CBA-EVs. This finding provides evidence that MRL-EVs can be an ideal inducing factor with excellent chondrogenic efficacy.

Extracellular vesicles contain many regulatory signals, such as proteins, RNAs, and microRNAs, which may play a key role in mechanism underlying their ability to reduce inflammation, induce tissue repair, and alter cellular signaling [37]. To further investigate the precise mechanism of extracellular vesicles in OA repair, we analyzed differences in the expression of miRNAs between CBA-EVs and MRL-EVs by miRNA-seq data analysis. In this study, we detected 180 significantly differentially expressed miRNAs (80 upregulated and 100 downregulated) in MRL-EVs compared with CBA-EVs. Many studies indicated that extracellular vesicles can regulate corresponding biological processes by affecting related pathways in receptor cells [38, 39]. We selected 20 differentially expressed miRNAs showing high fold change to predict their target genes and to perform pathway analysis. In our study, a majority of predicted miRNAs involved in signaling pathways were related to chondrogenesis and inflammatory regulation, including the Jak-STAT signaling pathway, Ras signaling pathway, FoxO signaling, chemokine signaling pathway, inflammatory mediator regulation of TRP channels, and MAPK signaling pathway. It is clear that differentially expressed miRNAs affected target genes which are mainly involved in the regulation of inflammation. These results, along with previous studies of MRL/MpJ mice, had different serum and synovial fluid IL-1β profiles after fracture and that macrophages from MRL/MpJ mice have lower upregulation of inflammatory cytokines [40]. The results of the GO analyses also revealed that certain differentially expressed miRNAs in extracellular vesicles are closely related to the chondrocyte proliferation and migration, such as FoxO signaling; are critical regulators of the fate of chondrocytes; and may have a protective effect during oxidative stress-induced chondrocyte dysfunction [41]. Ras signaling pathways are critical to induced proliferation and differentiation, the stress fiber assembly, and MAPK activation in BMSCs [42].

After validation by qRT-PCR, three miRNAs (miR-148a-3p, miR-221-3p, miR-222-3p) were observed to be significantly upregulated, while four miRNA (let-7b-5p, miR-22-3p, miR-125a-5p, miR-26a-5p) expression in MRL-EVs was decreased. Previous studies described miR-221 positively regulates chondrogenesis of chick limb MSCs [43]. Many studies indicated that by targeting *Pten* and *Timp3* tumor suppressors, miR-221&222 induced TRAIL resistance and enhanced cellular migration through activating the AKT pathway [44–46]. We found that the expression of miR-221- 5p was higher in MRL-EVs compared with CBA-EVs. From the miRNA-mRNA network, we found that *Pten* and *Timp3* genes were targeted by the miR-148a-3p and miRNA-221-3p, respectively, which revealed that these miRNAs may have functions in regulating chondrogenesis. In accordance with these published findings, we detected high level of miR-221-3p in MRL-EVs. In addition, we found that the CPC-EV-induced promotion of chondrogenesis function was partially attenuated by miR-221-3p inhibition. These findings suggest that miR-221-3p is one of the critical mediators in CPC-EV-induced promotion of chondrocyte proliferation and migration in vitro. Previous research has found that miR-22 inhibition suppresses MMP-13 expression, upregulates peroxisome proliferator-activated receptor-alpha (PPAR-α) and bone morphogenetic protein-7 (BMP-7) expression, and inhibits IL-1β expression in OA chondrocytes [47]. Additionally, our results partially confirmed that miRNA-22-3p targeted *Ppara*, *Max*, and *Arpc5*. In another study, MSC exosomal miR-125b overexpression was found to suppress IL-1β-induced upregulation of ADAMTS-4 in human OA chondrocytes [48]. These results were consistent with the results of our study about the downregulation of miR-22 and upregulation of miR-125b in MRL-EVs, indicating that miR-22 and miR-125b may play a role in regulating inflammatory and protecting MRL/MpJ against injury-induced cartilage damage. miRNA-125b-5p was predicted to have a high probability of binding to *Suv39h1*, *Tspan12*, *Bmf*, and *Bak1.* Very little is known about the functions of these genes, and they deserved further investigation.

Our results indicated that exosomal miRNAs may play a vital role in enhancing cartilage regeneration. Extracellular vesicles can be ingested more rapidly by homotypic cells, as opposed to other cells [49]. We believe that the combination of CPC-EVs will serve as excellent “inducing factors” to the repair of clinical OA. We should indicate that there are some limitations to this study. The detailed mechanisms of how miRNA 221-3p from CPC-EVs enhance migration and proliferation of chondrocyte require further exploration, and the further studies are urgently needed to explore the effects of exosomal miRNA 221-3p against OA in vivo. In addition, the specific molecular mechanisms of extracellular vesicles involving in OA repair remain elusive. Therefore, these issues should be explored in future studies.

## Conclusions

In this study, we investigated the role and underlying mechanisms of CBA-EVs and MRL-EVs on chondrocyte proliferation and migration in vitro and on OA in vivo. Our data revealed that CPCs could produce amounts of extracellular vesicles, which exhibited the typical morphological features of extracellular vesicles. The CPC-EVs ameliorated the OA severity in vivo and significantly stimulated chondrocyte migration and proliferation in vitro. QRT-PCR results confirmed the validity of differentially expressed miRNAs identified by RNA sequence. There are three miRNAs (miR-148a-3p, miR-221-3p, miR-222-3p) that were observed to be significantly upregulated while four miRNA (let-7b-5p, miR-22-3p, miR-125a-5p, miR-26a-5p) expression in MRL-EVs was decreased. MiR-221-3p was found to be highly enriched in MRL-EVs and served as a critical mediator in CPC-EV-induced regulation of function of chondrocytes in vitro. Our study provides evidence that intra-articular extracellular vesicles secreted by chondrogenic progenitor cells can prevent the development of OA. These results could help facilitate future research in the identification and development of novel approaches to treat OA.

## Data Availability

All data generated or analyzed during this study are included in this manuscript.

## Abbreviations

BMP-7: Bone morphogenetic protein-7
CBA-EVs: Extracellular vesicles secreted by CPCs from control CBA mice
CPCs: Chondrogenic progenitor cells
DMM: Destabilization of the medial meniscus
GO: Gene Ontology
KEGG: Kyoto Encyclopedia of Genes and Genomes
MRL-EVs: Extracellular vesicles secreted by CPCs from MRL/MpJ mice
MSCs: Mesenchymal stem cells
NTA: Nanoparticle tracking analysis
PPAR-α: Peroxisome proliferator-activated receptor-alpha
TEM: Transmission electron microscopy
